# Multiple nivolumab-induced CNS demyelination with spontaneous resolution in an asymptomatic metastatic melanoma patient

**DOI:** 10.1186/s40425-019-0818-3

**Published:** 2019-12-02

**Authors:** Vincent Pillonel, Vincent Dunet, Andreas F. Hottinger, Gregoire Berthod, Luis Schiappacasse, Solange Peters, Olivier Michielin, Veronica Aedo-Lopez

**Affiliations:** 1 Department of Medical Oncology, Lausanne University Hospital (CHUV) and University of Lausanne (UNIL), Lausanne, Switzerland; 2 Institute of Pathology and Medical Genetics, University Hospital Basel, Basel, Switzerland; 3 Department of Diagnostic and Interventional Radiology, Lausanne University Hospital (CHUV) and University of Lausanne (UNIL), Lausanne, Switzerland; 4 Department of Clinical Neurosciences, Lausanne University Hospital (CHUV) and University of Lausanne (UNIL), Lausanne, Switzerland; 5 Department of Oncology, Hôpital du Valais, Sion, Switzerland; 6 Department of Radiation Oncology, Lausanne University Hospital (CHUV) and University of Lausanne (UNIL), Lausanne, Switzerland; 7 Swiss Institute of Bioinformatics, University of Lausanne, Lausanne, Switzerland

**Keywords:** Immune checkpoint inhibitors, Nivolumab, Immune related adverse events, Neurological toxicities, CNS demyelination, Metastatic melanoma

## Abstract

**Background:**

Immune checkpoint inhibitors (ICPis) have revolutionised the treatment of melanoma by significantly increasing survival rates and disease control. However, ICPis can have specific immune-related adverse events, including rare but severe neurological toxicity.

**Case presentation:**

We report a 44-year-old man diagnosed with stage IIIB melanoma who developed metastatic disease (pulmonary and brain metastases) and was treated with stereotactic radiosurgery and nivolumab immunotherapy. He developed asymptomatic multifocal diffuse white matter lesions consistent with active central nervous system demyelination seen on brain MRI. One month after cessation of the immunotherapy, spontaneous regression of the demyelinating lesions was observed, suggesting a nivolumab-related toxicity.

**Conclusion:**

We report the first case of a melanoma patient with an asymptomatic and spontaneously reversible central nervous system demyelination following nivolumab immunotherapy. This case highlights the need for better recognition of such atypical and rare neurological toxicities which could be mistaken for progressive brain metastases. Early recognition and appropriate management are crucial to reduce severity and duration of these toxicities, especially for patients with less favourable evolution.

## Background

Immune checkpoint inhibitors (ICPis), ipilimumab and nivolumab, are recombinant human monoclonal antibodies which target cytotoxic T-lymphocyte-associated antigen-4 (CTLA-4) and programmed death-1 (PD-1) receptor, respectively. By blocking these key immune suppressive molecules on T cell surface, they elicit a potent immune response against cancer cells that managed to hijack these natural inhibitory signals [1]. Ipilimumab and nivolumab provide significant clinical benefits in patients with advanced melanoma [2–9] and multiple other tumor types, leading to FDA-approval of ipilimumab in 2011 and nivolumab in 2014 [1]. However, immunotherapies may elicit imbalances in immunologic tolerance which can result in excessive unregulated immune response with inflammatory or autoimmune side effects [10]. Hence, despite significant clinical benefit, the use of ICPis is frequently associated with a large spectrum of immune-related adverse events (irAEs) [2–9, 11], including rare but severe (grade 3–4) neurological toxicities [12–14]. Patients may develop a variety of neurological disorders including transient peripheral neuropathies, Guillain-Barré syndrome, myositis, myasthenia gravis, or less frequently central nervous system (CNS) toxicity such as hypophysitis, immune encephalitis, vasculitis, aseptic meningitis and multiple sclerosis. These neurological irAEs are yet extensively reviewed [12–15]. However, there has been only few scarce reports of CNS demyelination in association with ICPIs. One case was reported after nivolumab [16] and one after ipilimumab [17], which were both severe and eventually fatal. One more case of CNS demyelination resulting in neurological symptoms was reported after pembrolizumab, another PD-1 inhibitor [18]. Here, we present the first case of a melanoma patient with asymptomatic and spontaneously reversible CNS demyelination following nivolumab immunotherapy.

## Case presentation

A 44-year-old Caucasian man was diagnosed in March 2017 with a stage IIIB cutaneous nodular melanoma on the right forearm, with a tumor Breslow thickness of 3.43 mm, without ulceration (pT3a), one clinically detected tumor-involved axillary lymph node (pN1b), and no evidence of distant metastasis (cM0). He was treated with wide local excision, axillary lymph node dissection, and then with high-dose adjuvant ipilimumab monotherapy at 10 mg/kg i.v., according to EORTC 18071 protocol [7, 19]. Two days after the first ipilimumab infusion, he developed a persistent grade 2 colitis, which was corticosteroid-resistant, treated with infliximab, and that imposed termination of the treatment.

In September 2017, a follow-up computed tomography (CT) scan revealed pulmonary progression (one unique lesion) and wedge resection of segment 10 of the left inferior lobe was performed. The pathology confirmed metastatic melanoma, programmed death-ligand 1 (PD-L1) positive (60%) and wild-type *BRAF*. Three months later, subsequent imaging by CT scan and brain magnetic resonance imaging (MRI) revealed metastatic progression in lung with multiple lesions in the left superior and inferior lobe, hilar lymph nodes, and brain with one cerebellar and 4 millimetric contrast enhancing lesions in the frontal white matter. A CyberKnife (Accuray Incorporated, Sunnyvale, California) stereotactic radiosurgery (SRS) was administered 2 weeks later to the 5 cerebral lesions in one single fraction of 24 Gy and an immunotherapy anti-PD1 with nivolumab (3 mg/kg as monotherapy) was initiated. The decision to administer nivolumab as monotherapy was based on the very high PD-L1 positivity (60% of tumor cells), but also to minimize the risk of new irAEs, given his previous ipilimumab-induced corticosteroid-resistant colitis, and knowing that combination of ipilimumab and nivolumab result in more complications [3, 12, 13].

**Fig. 1:**
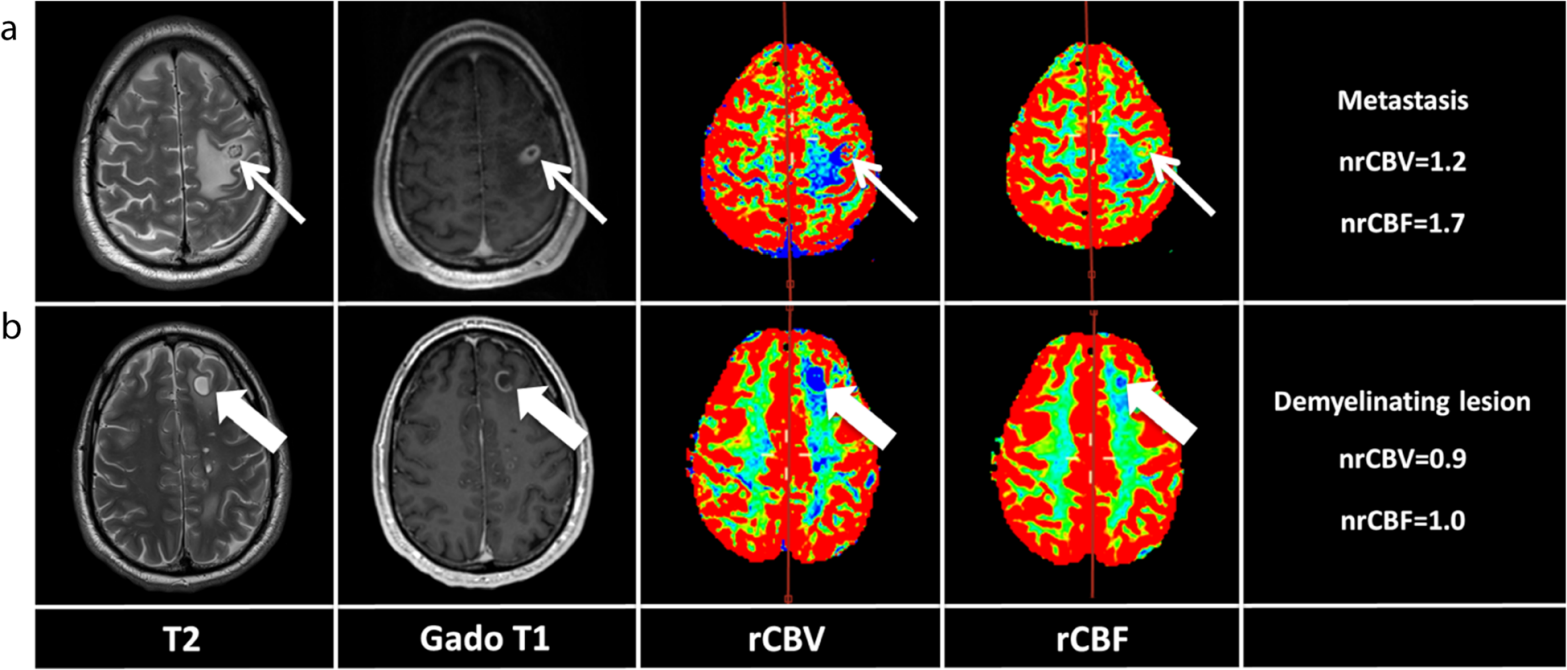
Comparison of the appearance of metastasis and demyelinating lesion in this patient. **a** The patient was treated by stereotaxic radiotherapy in January 2018 for several brain metastases, the largest being located at the cortico-subcortical junction of the left precentral gyrus. The lesion appeared hypointense with central necrosis and large perilesional oedema on T2-weighted images, had complete ring enhancement on post-contrast T1-weighted images, and increased normalized cerebral blood volume (nrCBV = 1.2) and cerebral blood flow (nrCBF = 1.7) ratios on perfusion weighted images. **b** In June 2018, he developed several demyelinating lesions, the largest being located in the left superior frontal gyrus. Contrary to the brain metastasis, the lesion was juxtacortical, had little perilesional oedema, partial ring enhancement (i.e. open ring sign), and low nrCBV and nrCBF

Two weeks after the first nivolumab infusion the patient presented with asthenia, headache, and apraxia of the upper right limb with impaired coordination of the right hand, and later developed a grade 1 erythematous maculopapular rash. A brain MRI showed multiple new metastatic brain lesions in the cerebellum, the left frontoparietal cortex, and the brain stem. The lesions were all complicated by perilesional oedema, for which he was administered dexamethasone (1 mg i.d. for 7 days and 5.25 mg tapered over 14 days). There was no evidence of infection and thyroid function studies were normal. Within 1 week, he presented at the hospital after a generalized epileptic seizure with clonic movements of the right-hand side of his body. Electroencephalogram (EEG) recording, performed after the seizure, was considered normal despite the presence of a discreet left temporal slowing. MRI revealed no changes in the known brain metastases and no evidence of ischemic or haemorrhagic events. He was hospitalized and an anti-epileptic treatment was introduced (Levetiracetam 500 mg bid) which prevented a recurrence of the seizures. In January 2018, CyberKnife SRS was administered to treat 7 new small metastasis (24 Gy in one fraction) and 3 large ones (35 Gy in five fractions) (Fig. 1a).

**Fig. 2:**
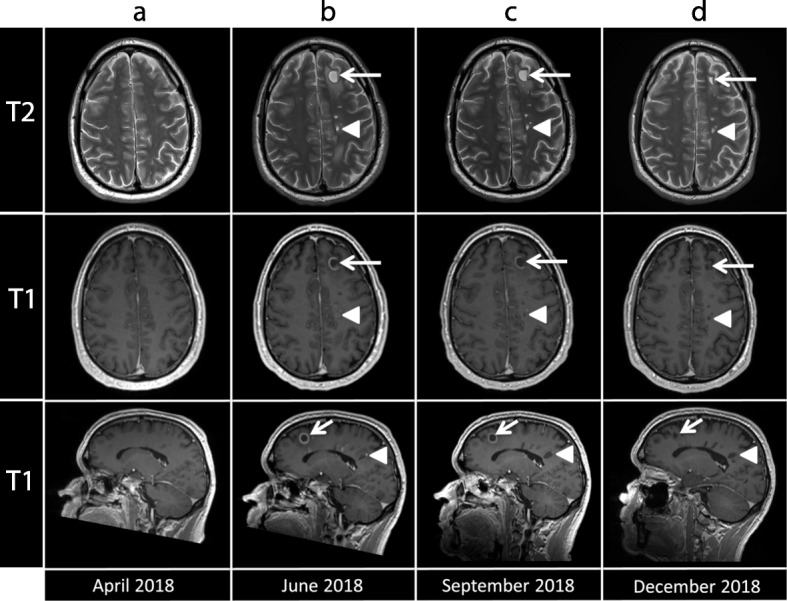
Display of demyelinating lesions evolution over 6 months. Successive brain MRI are displayed on axial T2-weighted images (top row), axial (middle row) and sagittal (bottom row) T1-weighted images after i.v. infusion of gadolinium contrast media. **a** Baseline in April 2018 before CNS demyelination. **b** Demyelinating lesions were diagnosed in June 2018. The largest lesion was located in the juxtacortical white matter of the left superior frontal gyrus (arrows) while numerous small ovoid lesions (arrows’ head) were located deep in the periventricular fronto-parietal white matter with a long axis perpendicular to the ventricle, corresponding to the typical “Dawson fingers” pattern. **c** After nivolumab discontinuation, lesions enhancement progressively decreased in September 2018. **d** The lesions disappeared in December 2018, leading to the characteristic “black hole” pattern on T1-weighted images

In February 2018, the immunotherapy with nivolumab was resumed (3 mg/kg, every 2 weeks). Follow-up brain MRI in April 2018 showed early evidence of good response with decrease in size or disappearance of the multiple pre-existing lesions without any new metastasis (Fig. 2a). In June 2018, after 11 cycles of nivolumab, a routine follow-up brain MRI showed multiple new diffuse white matter lesions, consistent with active CNS demyelination with a patient that was completely asymptomatic (Fig. 2b). These lesions consisted of multiple, well-defined, ovoid, T2-hyperintense lesions with incomplete ring enhancement (i.e. open ring sign) after i.v contrast administration and hypovascularization on perfusion weighted imaging (Fig. 1b). In addition, there was no abnormal diffusion restriction on diffusion-weighted imaging (DWI) in these lesions. They were mainly located in the juxtacortical and periventricular white matter of the fronto-parietal lobes, respecting Dawson fingers distribution, classical of demyelinating lesions (Fig. 2b). In contrast, previously treated brain metastases were T2-hypointense with central necrosis and large perilesional oedema, had complete ring enhancement on post-contrast T1-weighted imaging and hypervascularization on perfusion weighted imaging (Fig. 1a). Hence, demyelinating lesions could radiologically clearly be distinguished from brain metastases. The patient was asymptomatic and there were no findings indicating an infection or progression of his melanoma. Systematic neurological examination did not reveal any cranial or peripheral nerve abnormalities and he showed no cognitive function impairment. The EEG was repeated and was found unchanged from previous exams. Cerebrospinal fluid (CSF) analysis showed clear appearance with normal glucose and lactate levels. Elevated white blood cells (14 × 10^6^/l) and lymphocytes (13 × 10^6^/l) counts were found. Elevated protein level (594 mg/l, normal range: 150–450) and elevated albumin level (316 mg/l, normal range: 80–300) were observed. The CSF thus revealed a disrupted blood-brain barrier. Oligoclonal bands were absent, Immunoglobulins gamma (IgG) pattern and total IgG levels were normal in the CSF. Protein electrophoresis was normal, and serum autoantibody testing (anti-CNS, anti-LGT1, anti-CASPR2, anti-NMDA-R, anti-GluR1–2 AMPA) was negative. No tumor cell could be identified. Nivolumab immunotherapy was discontinued due to these demyelinating lesions. Since the patient was asymptomatic, it was decided not to give him any immunosuppressive treatment.

**Fig. 3:**
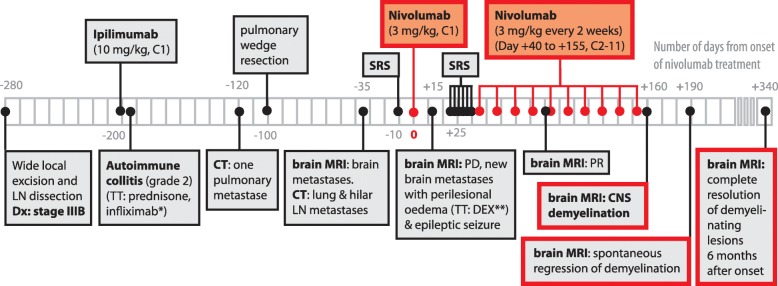
Timeline of the patient’s clinical, therapeutic, and radiological course. The 11 cycles of nivolumab administration and its related onset and resolution of CNS demyelinating lesions are highlighted in red. A time reference has been included, with Day 0 referring to the day of onset of nivolumab treatment. Abbreviations: LN: lymph node; C: cycle; SRS: stereotactic radiosurgery; Dx: diagnosis; TT: treatment; DEX: dexamethasone; PD: progressive disease; PR: partial response; MRI: magnetic resonance imaging; CT: computed tomography; CNS: central nervous system. *5 mg/kg; **1 mg i.d. for 7 days, and 5.25 mg tapered over 14 days

Strikingly, 1 month after cessation of nivolumab, the multiple demyelinating lesions spontaneously regressed (Fig. 2c), strongly suggesting an IrAEs of the immunotherapy, which was permanently discontinued. The patient has been followed by close monitoring for neurological symptoms and remained asymptomatic. Follow-up brain MRI every 3 months revealed complete resolution of these demyelinating lesions 6 months after initiation of nivolumab treatment (Fig. 2d and Fig. 3) along with stability in size and appearance of the prior identified cerebral metastases without new lesions. Tumor evaluation every 3 months by 18F-fluorodeoxyglucose (FDG) positron emission tomography (PET)/CT scan did not reveal any hypermetabolic lesions and confirmed a complete systemic and cerebral response over 12 months after occurrence of his irAE.

## Discussion and conclusions

Neurological complications of ICPis are rare, but often severe and may be life threatening, making its management challenging. This case study provides the first description of asymptomatic CNS demyelination after anti-PD1 blockade with nivolumab for metastatic melanoma with a spontaneous reversible course.

The patient reported here underwent an immunotherapy with nivolumab subsequent to adjuvant ipilimumab, which had to be discontinued after one dose of 10 mg/kg due to autoimmune colitis. Overall, the patient had been tolerating well the 11 cycles of nivolumab and was fully asymptomatic at the time of detection of lesions, radiologically compatible with CNS demyelinating lesions. Multiple juxta-cortical and periventricular white matter lesions with Dawson fingers distribution, open ring sign on post-contrast T1-weighted imaging and hypovascularization on perfusion weighted imaging were typical for demyelinating lesions.

Neurological adverse events of ICPis remain a complex diagnosis of exclusion [13]. In our case, all other differential diagnoses have been ruled out, including brain metastasis and leptomeningeal carcinomatosis for progressive oncologic disease, but also other demyelinating diseases of the CNS like multiple sclerosis as well as vascular, and infectious causes. The long interval of 12 months between the unique dose of ipilimumab and the occurrence of CNS demyelination argues against a role of this antibody. In previous case reports, the median time to onset of neurological irAEs following ICPis (mainly ipilimumab) was approximately 6 weeks (range: 1 to 74 weeks) and mostly occurs during the induction phase [20]. However, spontaneous regression of the radiological CNS lesions after nivolumab cessation strongly suggests a direct relationship between the two. Indeed, the onset and improvement of radiological lesions correlates with the administration and discontinuation of nivolumab, respectively. This, along with the absence of any other possible etiology, indicates that CNS toxicity is most likely nivolumab-related. Considering the asymptomatic course, nivolumab immunotherapy was discontinued without administration of immunosuppression to avoid dampening of the anti-tumor activity. Remarkably, even without treatment all demyelinating lesions completely resolved 6 months after nivolumab discontinuation.

So far, CNS demyelination in association with ICPis treatment have not been reported in large cohorts of patients [21, 22], but only in few isolated cases [16–18]. Unlike in the case reported here, they were all severe, symptomatic and not spontaneously reversible. In addition, in two of these case reports, patients had either clinical or radiographic evidence of preexisting multiple sclerosis flares. Interestingly, PD1-blockade was previously shown to worsen demyelinating disease in animal models of multiple sclerosis [23, 24]. Moreover, a PD-1 gene polymorphism was found to be associated with disease progression in multiple sclerosis patients [25]. Taken together, these pre-clinical studies and the CNS demyelinating toxicity of PD-1 inhibitors observed in 3 case reports including this case [16, 18], suggest that the PD-1 pathway may play a regulatory role in the development of CNS demyelination.

This case report highlights the need for better recognition of atypical and rare neurological toxicities such as CNS demyelination under anti-PD1 treatment. It is essential to recognize such lesions as they may be mistaken for progressive brain metastases. Early recognition and appropriate management are crucial to reduce severity and duration of these toxicities, especially for patients with less favourable evolution [13, 15]. Atypical neurological irAEs like CNS demyelination may be more prevalent than expected and their real incidence has been possibly underestimated due to lack of recognition and/or underreporting, as these irAEs could be transient [12] and possibly asymptomatic like reported in this case. Of note, patients with active brain metastases were excluded from most pivotal clinical trials and hence, such rare asymptomatic CNS adverse events may have been missed in this particular setting. It is important, that oncologists, neurologists and radiologists are aware of such atypical and rare neurological toxicities, which are anticipated to rise given the increased use of ICPIs to treat melanoma and other malignancies. Further clinical trials are needed to evaluate the exact neurological safety profile and clarify the risk-benefit ratio of these ICPis in order to determine optimal management guidelines.

## Data Availability

Available upon request.

## Abbreviations

CNS: Central nervous system
CSF: Cerebrospinal fluid
CT: Computed tomography
CTLA-4: Cytotoxic T-lymphocyte-associated antigen-4
FDA: Food and Drug Administration
FDG: 18F-fluorodeoxyglucose
ICPis: Immune checkpoint inhibitors
irAEs: Immune-related adverse events
MRI: Magnetic resonance imaging
PD-1: Programmed death-1
PET: Positron emission tomography
SRS: Stereotactic radiosurgery
